# RETRACTED: Lucas et al. Health Impact and Psychosocial Perceptions Among French Medical Residents During the SARS-CoV-2 Outbreak: A Cross-Sectional Survey. *Int. J. Environ. Res. Public Health* 2021, *18*, 8413

**DOI:** 10.3390/ijerph23091168

**Published:** 2026-09-07

**Authors:** David Lucas, Sandrine Brient, Bisi Moriamo Eveillard, Annabelle Gressier, Tanguy Le Grand, Richard Pougnet, Jean-Dominique Dewitte, Brice Loddé

**Affiliations:** 1 ORPHY Laboratory, University Brest, F-29200 Brest, France; brice.lodde@chu-brest.fr; 2 Occupational Health Service, Teaching Hospital, F-29200 Brest, France; sandrine.brient@chu-brest.fr (S.B.); moriamo.eveillard@chu-brest.fr (B.M.E.); annabelle.gressier@chu-brest.fr (A.G.); tanguy.legrand@chu-brest.fr (T.L.G.); richard.pougnet@chu-brest.fr (R.P.); jean-dominique.dewitte@chu-brest.fr (J.-D.D.); 3 Laboratoire d’Etude et de Recherche en Sociologie (EA 3149), Université de Brest—Bretagne Occidentale, F-29200 Brest, France

The journal retracts the article titled “Health Impact and Psychosocial Perceptions among French Medical Residents during the SARS-CoV-2 Outbreak: A Cross-Sectional Survey” [1], cited above.

Following publication, concerns were brought to the attention of the Editorial Office regarding the ethical approval of this study at the time the research was conducted.

In accordance with the journal’s standard procedures, the Editorial Office and Editorial Board conducted an investigation. During this process, the authors stated that ethical approval for the study was obtained retrospectively, after the research had commenced. As the research involved human participants and did not have appropriate ethical approval in place before the study was undertaken, the Editorial Board determined that this study did not meet the journal’s requirements or MDPI’s policy on research involving human participants. Consequently, the Editorial Board has decided to retract this article in accordance with MDPI’s retraction policy.

This retraction was approved by the Editor-in-Chief of the *International Journal of Environmental Research and Public Health*.

The authors have been informed of this retraction but did not provide a comment on this decision.
